# Systemic secukinumab treatment in patients with ankylosing spondylitis: the relationship between systemic and tear proinflammatory cytokines and ocular surface findings—a pilot study

**DOI:** 10.1007/s10067-025-07492-y

**Published:** 2025-05-20

**Authors:** Ozlem Dikmetas, Yasemin Kapucu, Semih Coşan, Nargiz Rustamova, Zehra Ozsoy, Sibel Kocabeyoglu, Umut Kalyoncu, Çağman Tan, İlhan Tezcan, Murat Irkec

**Affiliations:** 1 Department of Ophthalmology, Hacettepe University School of Medicine, Ankara, Turkey; 2 Division of Rheumatology, Department of Internal Medicine, Faculty of Medicine, Hacettepe University, Ankara, Turkey; 3 Hacettepe University Vasculitis Centre (HUVAC), Ankara, Turkey; 4 Department of Immunology, Erciyes University School of Medicine, Ankara, Turkey; 5 Department of Immunology, Hacettepe University School of Medicine, Ankara, Turkey

**Keywords:** Ankylosing spondylitis, Anti IL-17 receptor antibody, Dry eye disease, IL-17, Secukinumab

## Abstract

**Backgrounds:**

Dry eye disease (DED) is a very common ocular surface disease that affects millions of people around the world. When we look at the pathogenesis of the disease, it is seen that inflammation plays a role. Ankylosing spondylitis (AS) is the prototype of immune-mediated inflammatory rheumatoid diseases in the spectrum of axial spondyloarthropathies. In studies, many proinflammatory cytokines (TNF-α, IL-23, IFN-γ), especially IL-17, have been shown in the etiopathogenesis of AS. Recently, anti IL-17 receptor inhibitors (secukinumab) have been using. It selectively binds to the IL-17 receptor, preventing its association with the target receptor. From this point of view, it was aimed to evaluate the blood and tear proinflammatory cytokine levels and ocular surface parameters of the patients treated with secukinumab, before and during the treatment.

**Methods:**

This cross-sectional study included 12 patients with AS and 12 healthy individuals. The ocular surface and tear film were assessed using the Ocular Surface Disease Index (OSDI) questionnaire, tear film break-up time (TBUT), ocular surface staining, and Schirmer II test. The blood and tear samples were taken simultaneously. Signs and symptoms of DED were evaluated on the treatment day and 4 weeks, and 12 weeks after treatment. Tear and blood samples were taken once at the beginning of the study for the control group. Proinflammatory cytokine levels from collected tear and venous blood samples were examined in Pediatric Immunology laboratory. The tear levels of 30 cytokines were examined. Analyses were made with SPSS 25.0 package program.

**Results:**

Compared to controls, AS patients had higher OSDI (*p* = 0.01), similar corneal staining with fluorescein and lissamine green (*p* = 0.12), and lower TBUT (*p* = *0.04*). OSDI were found to be significantly different in the AS group at the measurement times (*p* = 0.01). IL-1B, IL-10, IL-13, IL-6, IL-12/IL-23p40, Rantes, Eotaxin, IL-17A, MIP-1A, GM-CSF, MIP-1B, MCP-1, IL-15, IL-5, IFN-G, IFN-A, TNF-A, IL-2, IL-7, IP-10, IL-2R, MIG, IL-4, and IL-8 levels in the AS serum and tears group did not differ at the first and third months (*p* > 0.05). IL-1RA measurements showed a significant decrease in the first and third months compared to baseline in the AS serum and tears (*p* = 0.04).

**Conclusions:**

The findings of this study showed that there was decreased IL-1RA in patients with AS after secukinumab treatment in tears and serum. Interestingly, lachrymal IL-17 levels were similar but not statistical significant changes with two ocular parameters TBUT and Schirmer’s test, suggesting a pathological role of IL-17 in rheumatological diseases. The results suggest that the inhibition of IL-1RA obtained by systemic administration of secukinumab but does not influence the severity of DED.

**Table:** 

**Key Points** *• Many proinflammatory cytokines (TNF-α, IL-23, IFN-γ), especially IL-17, have been shown in the etiopathogenesis of ankylosing spondylitis.* *• IL1 affects the local lymfoid nodes and IL 17 affects the lacrimal gland. IL-17 and its receptor are very important structures for the pathogenesis of inflammation.* *• Secukinumab is a monoclonal antibody that neutralize the IL 1β and IL 17 A.* *• The findings of this study showed that there was decreased IL-1RA in patients with ankylosing spondylitis after secukinumab treatment in tears and serum.*

## Introduction

Ankylosing spondylitis (AS) is a rheumatic disease which is an inflammatory and autoimmune. AS primarily effects the spinal and sacroiliac joints [1]. AS is also associated with some ophthalmic diseases. Acute anterior uveitis is the most common extraarticular ophthalmic finding [2]. Dry eye disease (DED) is also inflammatory disease with an increase in inflammatory cytokines [3]. Interleukin-1 (IL-1), IL-6, TNF-α, and IL-17 are the cytokines which increase with DED. IL1 affects the local lymfoid nodes and IL-17 affects the lacrimal gland. IL-17 and its receptor are very important structures for the pathogenesis of inflammation. IL-17 play role in proinflammatory activity and autoimmunity [4]. Previously studies usually were investigated in rheumatoid arthritis and sjogren syndrome (SS). The levels of IL-17 in tears were significantly increased in DED, which were associated with the SS severity. DED is characterized by a loss of homeostasis of the tear film accompanied by symptoms of ocular discomfort with potential defect to the ocular surface and visual function [5].

Secukinumab is a relatively new treatment option for AS [6]. Secukinumab is a monoclonal antibody that neutralize the IL-1β and IL-17 A. Recently, the use of biologic agents has been investigated for the treatment of DED, with the potential for preventing disease progression.

In this study, we evaluated the levels of inflammatory cytokines in tears and bloods of patients who treated with secukinumab due to AS.

## Materials and methods

This was a cross-sectional study conducted in a single tertiary referral academic center. The cases and controls were designed prospectively. The study was approved by the Institutional Review Board (KA-180138) and adhered to the tenets of the Declaration of Helsinki.

**Fig. 1:**
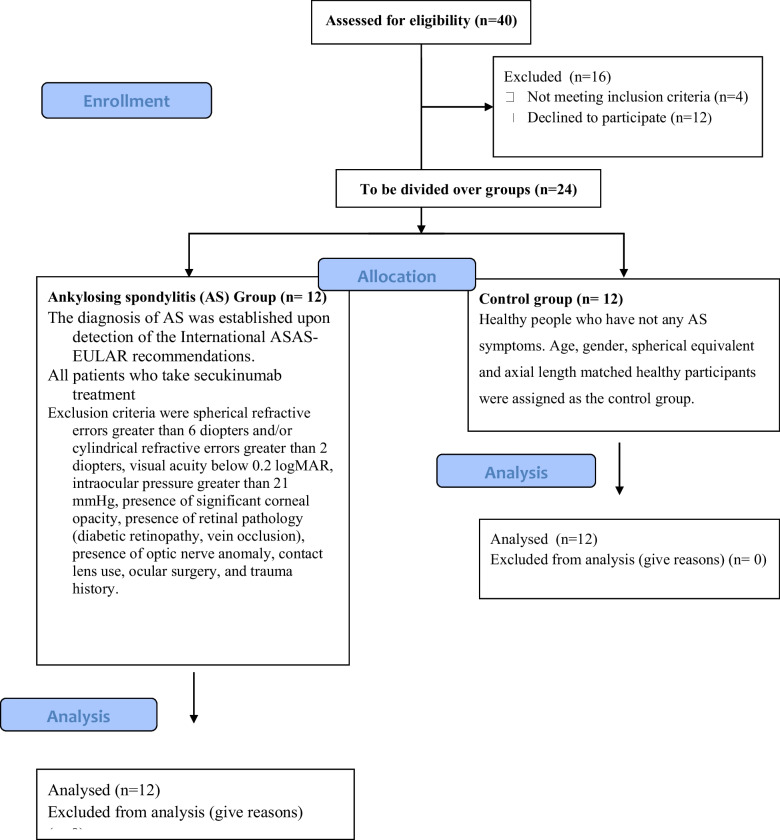
The CONSORT diagram illustrating the flow of participants throughout the study

The study cohort consisted of patients who is diagnosed with AS based on rheumatological findings and treated with secukinumab. The control group was selected from the healthy people who have not any AS symptoms. Age, gender, spherical equivalent, and axial length matched healthy participants were assigned as the control group (Fig. 1).

The diagnosis of AS was established upon detection of the International ASAS-EULAR recommendations [2]. Exclusion criteria were spherical refractive errors greater than 6 diopters and/or cylindrical refractive errors greater than 2 diopters, visual acuity below 0.2 logMAR, intraocular pressure greater than 21 mmHg, presence of significant corneal opacity, presence of retinal pathology (diabetic retinopathy, vein occlusion), presence of optic nerve anomaly, contact lens use, ocular surgery, and trauma history. Written informed consent was taken from all patients before study initiation. All patients underwent a detailed ophthalmic examination consisting of a best-corrected visual acuity assessment with a logMAR chart, slit-lamp biomicroscopy, and a dilated fundus examination. Ocular surface tests were made which are tear break up time (TBUT), ocular surface staining with fluorescein and lissamine green, and Schirmer’s test II (with anesthesia). The Schirmer II test was performed first, followed by a 10-min waiting period before performing other ocular surface tests such as TBUT and ocular surface staining. Ocular surface staining was graded with the Oxford scale from 0 to 5 [7]. The Ocular Surface Disease Index (OSDI, Allergan Inc.) questionnaire was done which was previously validated in Turkish [8]. The OSDI score was calculated using the following formula: OSDI = total score of the questions answered/25 (total number of questions). Tear and blood samples were taken control and AS patients. After the secukinumab treatment’s 1 month and third month, blood and tear samples were taken again from the AS patients group.

Tear and blood samples were collected from all participiants. Tear samples of 5 μL were collected from the tear meniscus of the lower fornix at the right eye with a pipette. Ten milliliters of venous blood sample was collected from an antecubital vein into EDTA tubes, samples were then centrifuged at 3000 rpm for 10 min, and the supernatants were stored in Eppendorf tubes at − 80 °C until assayed. The tear samples were placed in Eppendorf tubes, centrifuged at 6000 rpm for 5 min, and then stored at − 80 °C for study. For analysis, the tears were first diluted 1:10 in reagent diluents, then a bead-based immunoassay was used to analyze the concentration of 30 inflammatory molecules in the tear samples with a Luminex IS-100 device (Austin, TX, USA). A Human Cytokine 30-plex kit (Invitrogen; Waltham, MA, USA) measured the concentrations of granulocyte–macrophage colony-stimulating factor (GM-CSF), granulocyte colony-stimulating factor (G-CSF), interferon alpha (IFN-α), IFN-γ, IL-1β, IL-1RA, IL-2, IL-2R, IL-4, IL-5, IL-6, IL-7, IL-8, IL-10, IL-12, IL-13, IL-15, IL-17, TNF-α, monocyte chemoattractant protein-1 (MCP-1), interferon gamma-induced protein 10 (IP- 10), monokine induced by IFN-γ (MIG), RANTES, eotaxin, macrophage inflammatory protein-1 alpha (MIP-1α), MIP-1β, epidermal growth factor (EGF), fibroblast growth factor (FGF), hepatocyte growth factor (HGF), and vascular endothelial growth factor (VEGF). The results were analyzed with ProcartaPlex (eBioscience, USA) data analysis program.

## Statistical analysis

The distribution of the data was evaluated utilizing the Shapiro–Wilk test. Given the constrained sample size and the non-normal distribution observed in the majority of variables, non-parametric statistical tests were employed as the preferred analytical approach. Consequently, descriptive statistics are reported as the median and interquartile range (Q1–Q3) across all tables presented in the current study. In the study, Mann–Whitney *U* test analyses were performed to examine the differences in the demographic and ocular surface parameters between the control and patient groups, as well as the baseline tear cytokine levels. Friedman analyses were used to compare the baseline, 1st month, and 3rd month measurements of tear and serum cytokine levels within the same patient group. Bonferroni correction test was used to determine the measurements that make the difference in the results determined as the difference. *p* values less than 0.05 were considered statistically significant in the study. Analyses were performed using the SPSS Statistics version 25.0 software (IBM Corp., Armonk, NY, USA).

## Results

Twelve patients (7 M/5 F) with AS (mean age = 45.33 ± 8.41 years (range = 35–51 years)) and 12 (8 M/4 F) healthy control subjects (mean age = 39.42 ± 7.48 years (range = 30–45 years)) were included in the study (*p* = 0.13). The measurements of the lysamine green and fluorescein staining were not significantly different in the patient and control groups (*p* = 0.12, *p* = 0.12). The TBUT measurements were significantly lower in the AS group (7.5 ± 1.38 sn in the AS group and 10.58 ± 5.73 sn in the control group) (*p* = 0.04). Ocular surface survey measurements (OSDI) were found to be significantly higher in the patient group (*p* = 0.01). Lysamine green and fluorescein staining measurements observed that the initial, first month, and third month measurements were not significantly different in the AS patient group (*p* = 0.42, *p* = 0.45, respectively). OSDI scores were found to be significantly different in the patient group at the measurement times (*p* = 0.01) (Table 1). For Schirmer test, baseline, first month, and third month measurements were not significantly different in the AS patient group (*p* = 0.64). It was determined that the IL-1RA measurement was different in the groups and the measurements of the control and patient tear groups were significantly higher than the other groups (*p* = 0.01). It was determined that the IL-8 measurement was different in the groups and the measurements of the control serum group were significantly lower than the other groups (*p* = 0.02). The baseline level of cytokins of the control and patients’ tear and serum cytokin levels are summarized in Table 2. It was determined that the levels of the cytokins in the AS patients’ tears did not differ at the beginning of the study, at the first and third months (*p* > 0.05). However, a significant decrease was observed in IL-1RA levels in AS tears between baseline and the first month (*p* = 0.012), as determined by Bonferroni-adjusted pairwise comparisons following the Friedman test (Table 3). In AS serum samples, IL-1RA levels showed a significant increase from baseline to the first month (*p* = 0.008), according to post hoc analysis (Table 4). No other cytokines exhibited statistically significant changes across the three time points.

**Table 1:** Demographics of patients diagnosed with ankylosing spondylitis disease and control group

	Group		*p*
	AS (*N* = 12)	Control (*N* = 12)	
Age (year)	46.0 (38.0–50.5)	38.5 (32.75–45.0)	0.13
Visual acuity (logMAR)	1.00 (0.90–1.00)	1.00 (1.00–1.00)	0.74
Tear break up time (sn)	7.5 (6.0–8.75)	10.0 (5.25–16.25)	0.04*
Lissamine green staining	2.0 (1.0–2.0)	1.5 (1.0–2.0)	0.12
Fluorescein staining	2.0 (2.0–2.0)	1.5 (1.0–2.0)	0.12
OSDI	31.72 (15.62–46.58)	1.13 (0.00–10.60)	0.01*

Values are median (Q1–Q3). Differences were tested using the Mann–Whitney *U* test. *p ≤.05 was considered statistically significant

**Table 2:** The baseline level of control and patients groups’ tear and serum cytokine level

	Tears			Serum		
	Control	AS	*p*	Control	AS	*p*
IL-15	62.00 (60.25–68.00)	64.00 (59.00–67.50)	0.66	97.00 (49.75–178.00)	63.75 (49.50–79.00)	0.18
IL-5	8.00 (7.00–8.88)	7.00 (7.00–8.00)	0.41	7.50 (6.25–34.25)	7.50 (6.13–11.00)	0.76
IFN-G	15.00 (14.00–16.00)	14.00 (14.00–15.00)	0.25	12.75 (12.00–24.50)	13.00 (12.00–16.00)	0.93
IFN-A	8.25 (8.00–9.00)	9.00 (8.00–10.50)	0.03*	13.00 (9.50–54.63)	8.00 (8.00–15.50)	0.06
IL-1RA	2862.25 (1440.63–5514.75)	5229.75 (1299.00–9025.00)	0.01*	36.25 (29.50–102.75)	35.50 (30.00–44.38)	0.63
TNF-A	8.00 (7.00–9.00)	8.00 (7.25–8.75)	0.01*	15.00 (13.25–197.63)	19.50 (8.00–50.13)	0.29
IL-2	8.00 (8.00–9.00)	8.00 (8.00–9.75)	0.03*	15.00 (11.00–36.88)	14.50 (7.25–18.38)	0.35
IL-7	10.50 (10.00–13.00)	10.00 (9.00–19.75)	0.29	10.50 (9.25–12.75)	10.00 (8.63–10.00)	0.10
IP-10	71.00 (34.50–208.63)	160.00 (21.88–577.00)	0.02*	28.50 (17.88–39.00)	22.00 (11.00–45.50)	0.38
IL-2R	15.00 (13.00–16.00)	15.25 (13.00–18.75)	0.01*	49.50 (19.25–107.38)	29.50 (20.75–49.50)	0.48
MIG	24.00 (22.25–29.50)	27.50 (21.63–37.88)	0.91	24.00 (22.00–31.63)	24.50 (19.25–39.25)	0.89
IL-4	12.50 (12.00–14.00)	12.75 (11.00–14.63)	0.79	12.50 (11.00–13.75)	12.00 (9.25–17.75)	0.55
IL-8	213.50 (167.50–352.50)	452.25 (264.63–680.63)	0.02*	150.50 (123.75–196.00)	164.25 (123.25–228.75)	0.80
Total events	2639.00 (2568.25–2769.50)	2697.00 (2446.25–2983.25)	0.12	2779.00 (2649.00–2978.00)	2602.00 (2530.00–2764.00)	0.22

Values are median (Q1–Q3). Differences were tested using the Mann–Whitney *U* test. *p ≤.05 was considered significant

**Table 3:** Tear basal 1st and 3rd month measurement comparison

	Baseline AS tears	First month AS tears	Third month AS tears	*p*
IL-1B	9.55 (8.00–13.75)	10.00 (9.00–11.75)	10.00 (9.00–11.00)	0.95
IL-10	23.00 (23.00–25.75)	22.00 (21.25–24.50)	22.00 (21.13–24.00)	0.17
IL-13	9.00 (8.13–13.75)	9.00 (8.00–9.00)	8.25 (8.00–9.00)	0.17
IL-6	30.50 (20.75–37.88)	24.00 (20.25–48.25)	34.50 (19.25–61.13)	0.81
IL-12/IL-23p40	9.50 (7.25–14.38)	8.00 (7.63–11.38)	8.00 (7.25–9.00)	0.40
RANTES	138.00 (128.88–148.75)	137.50 (135.00–140.88)	135.00 (129.75–139.75)	0.29
Eotaxin	30.75 (26.50–33.63)	30.00 (28.00–31.00)	29.00 (26.50–31.75)	0.74
IL-17 A	11.25 (10.13–12.75)	10.50 (10.00–11.00)	11.00 (10.00–11.00)	0.23
MIP-1 A	9.50 (8.00–12.13)	9.00 (8.00–9.00)	9.00 (8.00–9.75)	0.29
GM-CSF	10.00 (10.00–12.00)	10.00 (10.00–11.00)	10.25 (10.00–11.00)	0.75
MIP-1B	11.25 (9.50–15.75)	10.00 (9.00–11.75)	9.50 (9.00–11.38)	0.25
MCP-1	10.50 (9.00–15.00)	9.50 (8.63–10.88)	10.00 (9.00–10.38)	0.37
IL-15	64.00 (59.00–67.50)	59.50 (57.25–64.00)	63.00 (61.63–64.63)	0.06
IL-5	7.00 (7.00–8.00)	8.00 (7.00–8.00)	7.00 (7.00–7.50)	0.34
IFN-G	14.00 (14.00–15.00)	14.00 (14.00–15.38)	14.50 (14.00–15.00)	0.98
IFN-A	9.00 (8.00–10.50)	8.25 (8.00–9.00)	8.00 (7.25–9.00)	0.59
IL-1RA	5229.75 (1299.00–9025.00)	3044.75 (2146.00–4475.00)	3054.50 (720.63–5940.13)	0.04*
TNF-A	8.00 (7.25–8.75)	7.50 (7.00–8.00)	8.00 (7.00–8.00)	0.24
IL-2	8.00 (8.00–9.75)	8.00 (7.13–8.00)	8.50 (8.00–9.00)	0.11
IL-7	10.00 (9.00–19.75)	11.00 (9.25–11.75)	10.50 (9.63–11.88)	0.93
IP-10	160.00 (21.88–577.00)	128.00 (22.25–636.75)	185.00 (17.25–359.13)	0.97
IL-2R	15.25 (13.00–18.75)	14.75 (12.25–16.00)	15.00 (14.00–15.88)	0.64
MIG	27.50 (21.63–37.88)	23.00 (21.25–32.75)	25.50 (22.25–30.00)	0.64
IL-4	12.75 (11.00–14.63)	12.00 (12.00–13.00)	12.00 (12.00–13.00)	0.92
IL-8	452.25 (264.63–680.63)	321.00 (143.88–684.63)	357.25 (146.75–652.25)	0.83
Total events	2697.00 (2446.25–2983.25)	2590.00 (2517.75–2706.50)	2670.00 (2622.50–2769.25)	0.38

Values are median (Q1–Q3). Differences were tested using the Friedman test. Pairwise post hoc comparisons were conducted using Bonferroni correction (adjusted significance level: *p* < 0.0167). Statistically significant results are marked with an asterisk (*)

**Table 4:** Comparison of the patient serum basal 1st and 3rd month measurements

	Baseline AS serum	First month AS serum	Third month AS serum	*p*
IL-1B	10.00 (8.25–25.00)	9.00 (8.00–62.88)	9.00 (7.10–10.00)	0.56
IL-10	22.50 (20.25–78.13)	26.00 (21.63–60.50)	26.00 (21.00–87.00)	0.93
IL-13	9.50 (8.00–12.00)	9.50 (7.00–11.75)	9.00 (8.00–11.00)	0.83
IL-6	49.00 (35.38–82.38)	36.00 (28.00–46.25)	34.50 (26.00–53.00)	0.24
IL-12/IL-23p40	195.00 (110.75–257.75)	160.25 (100.25–266.75)	122.00 (97.50–249.50)	0.76
RANTES	10,234.75 (9317.75–10,919.00)	10,436.50 (9692.50–10838.38)	10,415.00 (9878.00–11106.00)	0.68
Eotaxin	146.50 (98.50–289.00)	133.50 (87.38–274.75)	124.00 (71.00–248.00)	0.59
IL-17 A	9.50 (8.00–36.63)	8.50 (8.00–35.75)	10.00 (9.00–31.00)	0.94
MIP-1 A	10.25 (8.00–28.50)	16.50 (9.13–79.00)	12.00 (9.00–24.00)	0.51
GM-CSF	14.00 (9.13–37.50)	19.00 (9.00–37.75)	11.00 (9.00–73.50)	0.92
MIP-1B	60.00 (51.75–81.38)	60.50 (42.75–92.00)	61.00 (48.00–101.00)	0.99
MCP-1	42.50 (32.63–73.75)	64.75 (30.00–111.50)	38.00 (27.00–68.00)	0.61
IL-15	63.75 (49.50–79.00)	68.50 (50.25–84.25)	68.00 (49.50–73.50)	0.90
IL-5	7.50 (6.13–11.00)	6.00 (5.25–7.88)	6.00 (6.00–7.00)	0.17
IFN-G	13.00 (12.00–16.00)	12.00 (12.00–13.00)	13.00 (11.50–14.00)	0.41
IFN-A	8.00 (8.00–15.50)	9.50 (8.00–24.75)	9.00 (8.00–19.00)	0.60
IL-1RA	35.50 (30.00–44.38)	42.00 (26.00–79.50)	35.00 (23.00–51.50)	0.03*
TNF-A	19.50 (8.00–50.13)	15.00 (7.25–43.75)	16.00 (8.00–31.50)	0.75
IL-2	14.50 (7.25–18.38)	13.00 (8.25–41.25)	14.00 (9.00–22.00)	0.72
IL-7	10.00 (8.63–10.00)	9.50 (8.00–10.75)	9.00 (9.00–11.00)	0.77
IP-10	22.00 (11.00–45.50)	13.00 (11.25–65.00)	17.50 (11.00–26.00)	0.96
IL-2R	29.50 (20.75–49.50)	26.50 (21.00–58.75)	29.00 (21.00–52.00)	0.96
MIG	24.50 (19.25–39.25)	21.00 (20.00–65.50)	20.00 (20.00–26.50)	0.67
IL-4	12.00 (9.25–17.75)	11.00 (10.00–44.25)	12.00 (10.00–15.00)	0.91
IL-8	164.25 (123.25–228.75)	178.00 (126.13–262.00)	181.00 (138.00–222.00)	0.80
Total events	2602.00 (2530.00–2764.00)	2833.00 (2743.25–2913.50)	2552.00 (2474.00–2753.00)	0.10

Values are median (Q1–Q3). Differences were tested using the Friedman test. Pairwise post hoc comparisons were conducted using Bonferroni correction (adjusted significance level: *p* < 0.0167). Statistically significant results are marked with an asterisk (*)

## Discussion

In this study, we found that AS patients have higher OSDI but similar ocular surface findings. Serum and tear cytokine levels did not change after first and third month of treatment. IL-1RA measurements showed a significant decrease in the first and third months in the AS serum and tears. In previous placebo-controlled, double-blind studies, ocular surface staining, OSDI, TBUT, and visual acuity were measured in blood samples at the 1st, 4th and 8th weeks in patients receiving secukinumab [4, 9]. Drug levels were also checked. However, in these studies, the effectiveness of the drug on the eye surface could not be demonstrated. It has been shown that the reason for this may be that the drugs may not reach sufficient concentration in tears. In our study, IL-1RA level decreased in both tears and serum after treatment. However, other inflammatory cytokines did not change. The fact that it showed the same change in tears and blood suggests that secukinumab has similar effects on tears. Studies have shown that IL-1RA level may be associated with pain in Sjögren’s syndrome [10]. For this reason, IL-1RA, whose change was detected in our study, is of clinical importance. Amparo F et al. evaluate the evaluate the efficacy of treatment with the topical IL-1 receptor antagonist in patients with DED [11]. They found that treatment with topical IL-1 receptor antagonist, 2.5%, for 3 months was significantly reduced symptoms in patients with DED. These data suggest that the use of an IL-1 antagonist may have a therapeutic effect for patients with DED [12]. Sankar V et al. studied the efficacy of etanercept in the treatment of Sjögren’s syndrome [13]. There were no significant differences between the groups for changes in subjective measures of ocular symptoms and Schirmer I test. Similarly with our study, that study involved a systemically administered inhibitor of a proinflammatory cytokine. Also, like our study, there were minimal effects observed in the ocular findings. Proinflammatory cytokines inhibit lacrimal gland secretion. Immune-mediated destruction of the epithelial cells, due to the progressive inflammatory cell infiltration of the lacrimal glands, is responsible for the decline in tear. So IL-1 is very important for immmunological diseases; in this study, we aimed the effect of secukinumab to the tear cytokines.

Also longer follow-up is important for these patients because of the inflamation. For example, Papp KA et al. evaluate the efficacy and safety of different doses of secukinumab, anti-IL-17 A monoclonal antibody, in patients with moderate-to-severe plaque psoriasis [14]. They showed that response achievement was taken after 12 weeks, in moderate-to-severe psoriasis.

Some significant changes in AS cytokines were detected with secukinumab treatment. In the study conducted by Baeten D et al. in comparison with placebo, the speed of reaching the Assessment of Spondylo Arthritis International Society criteria for improvement (ASAS20) criteria and the effectiveness of the treatment were investigated [9, 15, 16]. It was found that 60% of the patients achieved the target treatment result within 6 weeks and that it was more effective than placebo [9]. Grosskreutz CL and colleagues examined the change in dry eye symptoms and signs with the use of canakinumab or secukinumab. In a randomized, double-blind, placebo-controlled study in 72 patients with moderate or severe dry eye, dry eye symptoms and signs were evaluated before treatment, at week 1, week 4, and week 8. Corneal staining, Schirmer test, TBUT, conjunctival hyperemia, OSDI score, visual acuity, and lubrication need were examined in the study. Corneal staining and other parameters did not change significantly in the 4 th week in all three groups. This study concluded that the use of systemic anti-inflammatory neutralizing drugs was not very effective on dry eye symptoms [17]. In our study, no significant change was detected in dry eye clinical dye and tear levels. However, OSDI scoring showed a significant change after treatment. This suggests that systemic secukinumab treatment is clinically effective. Dry eye syndrome and AS are inflammatory diseases that develop with similar etiopathogenesis and frequently occur together [4, 18, 19]. Considering these new treatment strategies that have emerged recently, systemic treatment is expected to have effects on dry eye syndrome and AS findings [20].

This study will be a guide in the approach to the treatment of diseases with inflammatory processes in terms of elucidating the approach to etiopathogenesis in future dry eye studies. In addition, the effectiveness of secukinumab treatment, which is frequently used, on dry eye has been shown for the first time in the literature. Therefore, it will make a significant contribution to the literature.

Our results should be interpreted in light of the study’s potential limitations. A major limitation is the relatively limited sample size. The main strength of the study is that only patients with AS treated with secukinumab were included, and as such, the results can be generalized to this subset of patients. The different severity of AS disease, and also the onset time of other rheumatological disease should be evaluated in future studies. We did not investigate the effect of different AS treatments The current study results suggest that further prospective studies with larger population sizes are recommended to assess the effects of AS.

In conclusion, the findings of this study showed that there was decreased IL-1RA in patients with AS after secukinumab treatment in tears and serum. Interestingly, lachrymal IL-17 levels were similar but not statistical significant changes with two ocular parameters TBUT and Schirmer’s test, suggesting a pathological role of IL-17 in rheumatological diseases. The results suggest that the inhibition of IL-RA obtained by systemic administration of secukinumab but does not influence the severity of DED.

## Data Availability

Data will be made available on request.
